# The significance of the increased expression of phosphorylated MeCP2 in the membranes from patients with proliferative diabetic retinopathy

**DOI:** 10.1038/srep32850

**Published:** 2016-09-12

**Authors:** Xiaohua Li, Xiaohui Liu, Haoyi Guo, Zhaoxia Zhao, Yun Sui Li, Guoming Chen

**Affiliations:** 1 Henan eye Institute, Henan key laboratory of keratopathy, Zhengzhou, China; 2 Henan Eye Hospital, Zhengzhou, China; 3 Department of Ophthalmology, Henan Provincial People’s hospital, Zhengzhou, China; 4 Department of Ophthalmology, Zhengzhou University People’s hospital, Zhengzhou, China.

## Abstract

The purpose of this study was to evaluate the correlation of expression of phosphorylated methyl-CpG binding protein 2-Ser421 (MeCP2-S421) and VEGF in the membranes of patients with PDR. We examined the expression of phospho-MeCP2-S80, S421, VEGF and PEDF in surgically excised PDR membranes from 33 patients with diabetes, and idiopathic epiretinal membranes from 11 patients without diabetes, using immunohistochemistry and western blot. The colocalization of MeCP2-S421 with VEGF, PEDF, CD31, GFAP and αSMA was revealed by fluorescent double labeling. The effect of CoCl2 and knock down MeCP2 using specific siRNA on the expression of MeCP2 and VEGF were analyzed in HUCAC cells by Western blot. We found that phospho-MeCP2-S421 was significantly increased in the membranes from the patients with PDR compared with the specimens from patients without diabetes (*P* < *0*.*01*). The expression of phospho-MeCP2-S421 was much stronger than that of phospho-MeCP2-S80 in the PDR membranes. Double labeling showed that the high phospho-MeCP2-S421 expression was associated with strong expression of VEGF, but not PEDF. Further, phospho-MeCP2-S421 and VEGF were increased by the stimulation of CoCl2 and knock down MeCP2 inhibited the expression of VEGF. Our result suggests that phospho-MeCP2-S421 might involve in the pathogenesis of PDR.

Diabetic retinopathy (DR) is a leading cause of blindness around the world^1^. The severe vision loss that occurs in proliferative diabetic retinopathy (PDR) is due to retinal neovascularization, hemorrhage, and the formation of fibrovascular membranes with subsequent tractional retinal detachment^2^,^3^,^4^. Specimens from patients with PDR reveal that a variety of locally produced factors, such as cytokines and growth factors, participate in the initiation and progression of the disease^3^,^5^. Vascular endothelial growth factor (VEGF) is believed to play a key role in the development of retinal angiogenesis in PDR^6^,^7^,^8^. The chronic hyperglycemia of diabetes may induce VEGF up-regulation via the PLA2/COX-2/VEGF-A pathway^9^,^10^, the PKCbeta/HuR/VEGF pathway^11^, the ERK1/2/COX-2/PGE2 pathway^12^ and the TLR2 and TLR4 pathway^13^. Increased VEGF levels and decreased levels of pigment epithelium-derived factor (PEDF) were revealed in specimens from eyes with PDR^6^,^7^,^8^. Although anti-VEGF therapy is an effective treatment for DR and PDR, the fact that some PDR patients do not benefit from the therapy^14^,^15^,^16^ suggests that PDR is a complex, multifactorial mediated retinal disease and that other mechanisms may participate in the pathogenesis of PDR.

Recent studies show that epigenetic alterations influence several common pathologic responses, including inflammation, ischemia, neovascularization, neoplasia, and neurodegeneration^17^,^18^,^19^. Importantly, epigenetic mechanisms may have a pathogenic role in diabetes, as well as in intraocular neovascularization, such as choroidal neovascularization and DR^20^,^21^,^22^,^23^,^24^,^25^. Accumulating evidence suggests epigenetic mechanisms such as DNA methylation, histone modifications and non-coding RNAs play important roles in the pathogenesis of retinal angiogenesis, including diabetic retinopathy. Patients with diabetes have shown a strong association between the polymorphism in SUV39H2, the gene that encodes histone methyltransferases, and retinopathy^26^. Altered histone acetylation machinery (histone acetyltransferases and histone deacetylases) is observed in the *in vitro* and *in vivo* models of diabetic retinopathy^27^. Models of diabetic retinopathy have shown an association between downregulation of miR-126, miR-146a and miR-200b and upregulation of VEGF, and between upregulation of miR-29b and protection of the retinal ganglion cells from apoptosis^28^,^29^. Notably, epigenetic drugs such as 5-aza-dC and Trichostatin A have been showed to increase PEDF expression and suppress the production of VEGF, ICAM-1, IL- 1β and MMP2^30^. Furthermore, in a rat model even hyperglycemia is terminated while the pathological process continues in the retina, which refers to abnormal of epigenetic mechanism^31^. However, phosphorylated methyl-CpG binding protein 2 (MeCP2)-Ser80 and Ser421 have not been studied in the pathogenesis of PDR, especially not in human specimens.

In the last 10 years, MeCP2 has been found to regulate a number of physiological and pathological conditions, such as development, cell proliferation and differentiation^32^,^33^,^34^, tumorigenesis, and neuronal and degenerative diseases^35^,^36^. MeCP2 associates with various transcription factors to form a complex, thereby regulating certain gene expressions^32^,^33^,^34^. MeCP2 is ubiquitously expressed in the mammalian central nervous system, and MeCP2 expression in the retina has been demonstrated^37^. Previous studies have shown that MeCP2 not only functions as a transcription suppressor but also enhances the expression of other genes, specifically through MeCP2 phosphorylation^38^,^39^. In the nervous system, MeCP2 phosphorylation activated by extracellular signals dynamically regulates gene expression^38^,^39^. In particular, the gene suppression of the brain-derived neurotrophic factor (BDNF)is reactivated by MeCP2 phosphorylation at Ser421^40^. Further, MeCP2-S421 phosphorylation is linked to cell growth signals in adult neuroprogenitor cells by the activation of aurora kinase B^41^. In contrast, MeCP2 Ser 80 phosphorylation inhibits activation of certain genes^42^. Interestingly, previous publications show that MeCP2 is an important modulator of VEGF expression in carcinoma cells and human endothelial cells^43^,^44^. These studies highlight the relevance of MeCP2, and especially of its phosphorylation at Ser421, to neovascularization^43^,^44^,^45^.

Little is known about the role of phosphorylated MeCP2 in diabetic retinopathy. In the present study, we examined the expression of phosphorylated MeCP2-S80, -S421, VEGF, and PEDF in the retinal membranes of patients with PDR and in epiretinal membranes from patients without diabetes. Our results provide first evidence that phosphorylated MeCP2 might involve in the pathogenesis of PDR.

## Results

### Patient information

The idiopathic epiretinal membrane (IEM) group consisted of 7 men and 4 women (average age, 44 years; range, 30–60 years.) The PDR group included 17 men and 16 women (average age, 47.6 years; range, 21–75 years) (see supplementary Table 1). No difference in gender distribution was noted.

### The expression of phosphorylated MeCP2-S421, S80 and non-phosphorylated MeCP2

**Figure 1:**
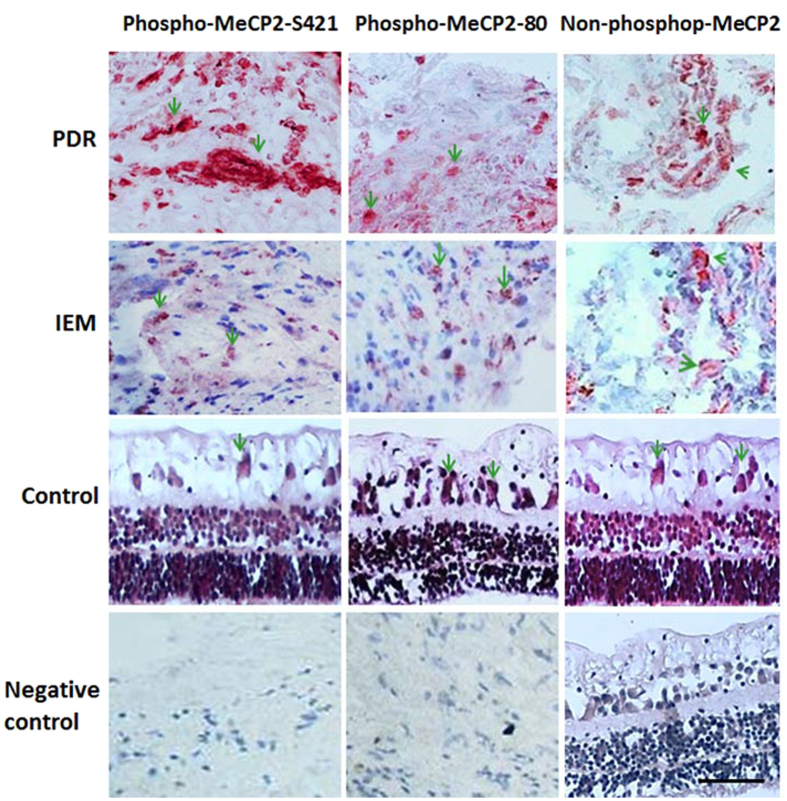
Immunohistochemical analysis of the expression of phospho-MeCP2-S421, -S80 and non-phospho-MeCP2 in human proliferative diabetic retinopathy membranes, idiopathic epiretinal membranes and normal retinal section. Red: positive staining for MeCP2; blue: hematoxylin contrast staining. All of arrows indicated positive MeCP2 staining in representative photomicrographs. Scale bar: 50 μm. Original magnification, 400×.

Most of the PDR membranes were moderately (12%) to intensely (85%) stained for phosphor-MeCP2-S421. Only 3% of the PDR membranes showed mild positive staining for phospho-MeCP2-S421 (supplementary Table 2), whereas 91% of the PDR membranes were mildly stained for phospho-MeCP2-S80. The difference in the staining intensity of phospho-MeCP2-S421 compared with phospho-MeCP2-S80 in the PDR membranes was significant (*P* < 0.001, supplementary Table 2). In the comparison of phospho-MeCP2-S421 staining in PDR membranes with the expression of phospho-MeCP2-S421 in IEM, 32 (97%) PDR membranes were moderately to strongly stained for phospho-MeCP2-S421, whereas most of the IEM (82%) were only mildly stained, the difference was markedly significant (supplementary Table 2; *P* < 0.001). In terms of the staining of phospho-MeCP2-S80, 30 (91%) patients with PDR and 10 (91%) patients with IEM were all mildly positive. No statistical difference was found in the expression of phospho-MeCP2-S80 between the two types of membranes (supplementary Table 2; *P* = 0.20). Notably, the intensity of expression of phospho-MeCP2-S80 and phospho-MeCP2-S421 in the IEM were similar; there was no significant difference (supplementary Table 2; *P* = 1). No significant difference was seen in the expression of non-phosphorylated MeCP2 in the membranes of PDR and IEM (Fig. 1).

Under higher magnification, it was found that the MeCP2 immunoreativity was localized in cytoplasm and nuclei, while VEGF positive staining was demonstrated in cytoplasm (S Fig. 1). In addition, the positive staining of phospho-MeCP2-S421 was abolished by preincubation of the phospho-MeCP2-S421 antibody with its specific blocking peptide; the result was shown in supplementary Fig. 2.

**Figure 2:**
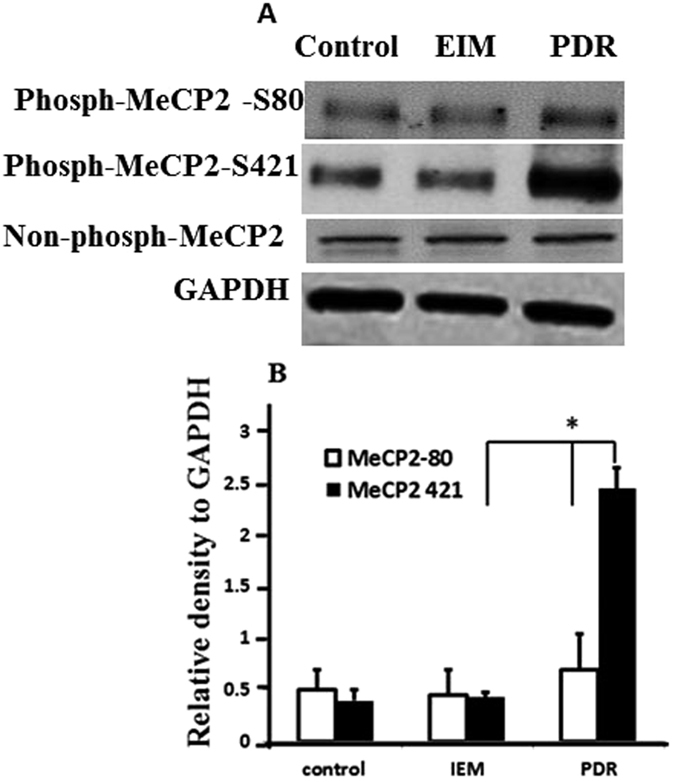
Western blot analysis of the proteins expression of phospho-MeCP2-S80, S421 and non-phospho-MeCP2 from paraffin sections of proliferative diabetic retinopathy membranes and idiopathic epiretinal membranes. (**A**) The extracted proteins were used in immunoblotting to detect phospho-MeCP2-S80, S421 and non-phospho-MeCP2. GAPDH was used as protein loading control. Blot is representative. (**B**) Relative levels of phospho-MeCP2-S421 and S80 from 3 independent experiments were quantified by measuring band intensity with Image J software. Data were normalized by comparing to the control. *P* *< 0.01 vs. control.

Western blot showed that phospho-MeCP2-S421 was markedly increased in PDR membranes in comparison with the expressions of phospho-MeCP2-S80 and phospho-MeCP2-S421 in IEM (Fig. 2A,B; *P *< *0*.*01*); no significant differences were found in the expression of phospho-MeCP2-S80 between PDR and IEM by Western blot assay (Fig. 2).

### Double labeling phosphorylated MeCP2-S80 and -S421 with VEGF and PEDF

**Figure 3:**
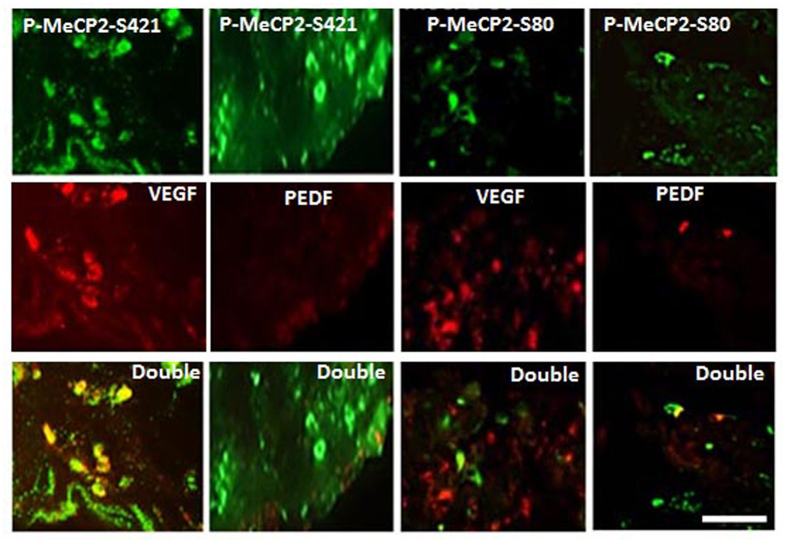
Phospho-MeCP2-S421 and S80 double labeling with VEGF and PEDF in human proliferative diabetic retinopathy membrane. The phospho-MeCP2-S421 and -S80 were stained as green. VEGF and PEDF were stained in red. Yellow shows co-localization of phospho-MeCP2-S421 and -S80 with VEGF or with PEDF. phospho-MeCP2-S421 was highly colocalized with VEGF. Scale bar: 100 μm. Original magnification, 200×.

**Figure 4:**
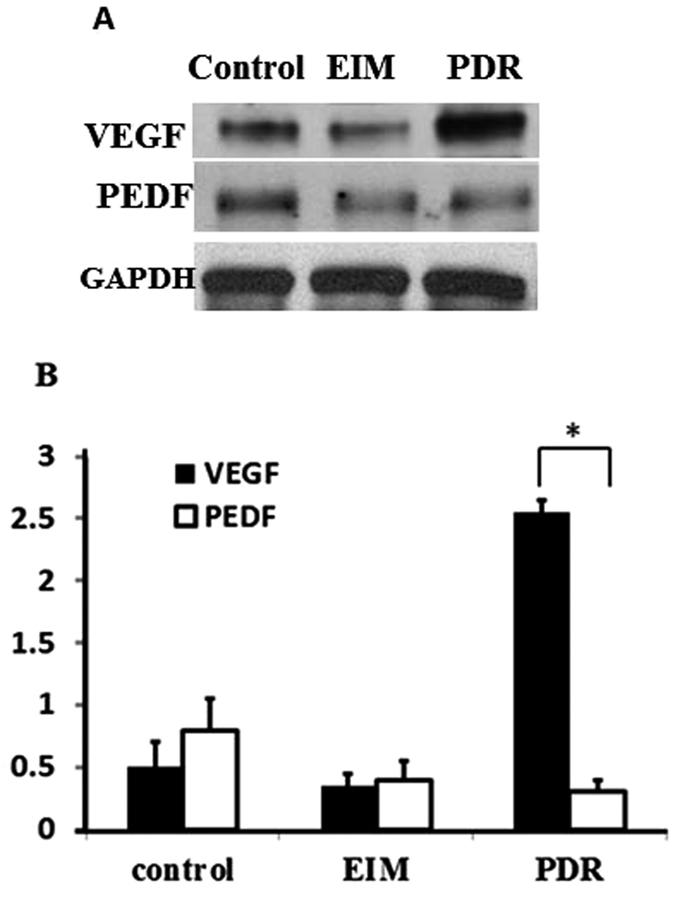
Western blot analysis of VEGF and PEDF expressed in idiopathic epiretinal membranes and proliferative diabetic retinopathy membranes. (**A**) The extracted proteins were used in immunoblotting to detect VEGF and PEDF. GAPDH was used as protein loading control. Blot is representative. (**B**) Relative levels of VEGF and PEDF from 3 independent experiments were quantified by measuring band intensity with Image J software. Data were normalized by comparing to the control. *P* *< 0.01.

Because the expression of phospho-MeCP2-S80 and phospho-MeCP2-S421 in IEMs was low and there was no difference in the expression of phospho-MeCP2-S80 and -S421 in the IEM membranes, all double labeling was performed in PDR membranes. Abundant phosphor-MeCP2-S421 was associated with strong VEGF expression (Fig. 3); importantly, the intensity of double labeling of phospho-MeCP2-S421 with VEGF was significantly higher than that of phospho-MeCP2-S80 double labeling with VEGF (supplementary Table 3; *P* < 0.01), and only a few phospho-MeCP2-S421-positive cells were colocalized with PEDF in PDR membranes. No difference was found between the double labeling of phospho-MeCP2-S80 with VEGF or PEDF in the PDR membranes (Fig. 3 and supplementary Table 2; *P* < 0.05). The reduced PEDF and increased VEGF expression in the PDR membranes were also demonstrated by Western blot (Fig. 4; *P *< *0*.*01*).

### Double labeling phosphorylated MeCP2-421 with CD31, GFAP and αSMA

We found no increased expression of phospho-MeCP2-S80 in either IEM or PDR membranes, and importantly, phospho-MeCP2-S421 was dominantly colocalized with VEGF. Therefore, we focused only on the colocalization of phospho-MeCP2-S421 with the cell markers such as CD31, GFAP and αSMA often found in PDR membranes. The results showed that the most prominent colocalization of phospho-MeCP2-S421was with CD31 (endothelial cell marker) (see supplementary Fig. 3), followed by the double labeling of phospho-MeCP2-S421 with GFAP (supplementary Fig. 3) in the PDR specimens. In contrast, only a few minor incidents of positive double labeling of phospho-MeCP2-S421 and αSMA were revealed in the membranes.

### CoCl2 induced expressions of MeCP2 and VEGF

Exposure of HUCAC cells to CoCl2 induced a significantly increases of the expression of phospho-MeCP2-S421 and VEGF as demonstrated by western blot (S Fig. 4), the maximal upregualtions of the expression of phospho-MeCP2-S421 and VEGF were seen at 24 hrs after addition of CoCl2 (*P* < 0.05); In contract to the increased expression of phospho-MeCP2-S421, the expression of phospho-MeCP2-S80 was reduced in the presence of CoCl2 (S Fig. 4, *P *< *0*.*05*).

### The effects of knock down MeCP2 on the expression of VEGF

It was found that knock down of MeCP2 using MeCP2 siRNA suppressed the expression of VEGF (S Fig. 5) significantly compared with cells transfected with scrambled siRNA in HUCAC cells (*P* < 0.05). The efficiency of reduction of MeCP2 gene was also revealed by the western blot analysis (S Fig. 5, *P *< 0.01).

## Discussion

Retinal neovascularization and subsequent traction retinal detachment are the major pathological processes leading to vision loss in advanced PDR^1^. VEGF is a key pro-angiogenic factor in the induction of retinal angiogenesis in PDR^6^,^7^,^8^; however, the molecules associated with VEGF in the formation of PDR are still under investigation. Our current study showed that phospho-MeCP2-S421 is abundant and its expression is much stronger than that of phospho-MeCP2-S80 in the membranes of PDR. Importantly, the increased expression of phospho-MeCP2-S421 was associated with strong expression of VEGF rather than of PEDF in the PDR membranes. Further, the cells that express phospho-MeCP2-S421 in the PDR membranes were also identified. The most prominent phosphorylated phospho-MeCP2-S421-bearing cells were CD31 positive cell, which was strongly colocalized with VEGF, as demonstrated by double immunostaining. The second major cell marker present in the PDR membranes and also colocalized with phospho-MeCP2-S421 was GFAP positive cells (indicating Müller and glia cells). αSMA-positive stroma cells were also detected; but only a few αSMA positive cells were double labeled with phospho-MeCP2-S421. Those cells are the principal cell types found in the PDR membranes in current research and have been reported to express VEGF^5^,^7^,^15^. Importantly, the cells express phospho-MeCP2-S421. Thus, our results suggest that phospho-MeCP2-S421 might play a role in the pathogenesis of PDR.

MeCP2 is found to globally bind across the genome in the methylated CpG dinucleotides. The loss of MeCP2 results in the upregulation or downregulation of many genes^32^,^33^,^38^,^39^; the removal of MeCP2 from CpG islands may result in the removal of gene repression and may enable the binding of transcriptional activators to gene promoters^32^,^33^,^38^,^39^. On the other hand, gene activation mediated by MeCP2 is through the phosphorylation of MeCP2; specifically, phosphorylated phospho-MeCP2-S421 may be permissive for changes in transcriptional regulation rather than for inhibition of gene activation^41^. Thus, MeCP2 phosphorylation is thought to affect gene transcription, neuronal morphology, synapse formation and animal behavior^33^,^38^,^39^,^41^. More importantly, it participates in the regulation of the expression of VEGF^43^,^44^. Here, we provide the evidence that MeCP2-S421 phosphorylation is associated with VEGF expression in PDR membranes. Moreover, our results show that increased phosphorylated MeCP2-S421 expression was correlated with enhanced expression of VEGF but not with phospho-MeCP2-S80. These data suggest that local MeCP2-S421 phosphorylation may participate in the regulation of VEGF expression in PDR. Notably, our results show the relevance of MeCP2 phosphorylation to VEGF expression as shown by other systems^43^,^44^.

In the current study, we demonstrated that the expression of phospho-MeCP2-S421 was more prominent than that of phospho-MeCP2-S80 in PDR membranes. Further, exposure HUCAC cells to hypoxia induced by CoCl2 results in increased expression of phospho-MeCP2-S421 and VEGF not phospho-MeCP2-S80. It is known both MeCP2 and VEGF are upregulated in hypoxia/ischemia condition^46^,^47^,^48^ importantly, we found that knock down MeCP2 using siRNA inhibited VEGF expression. Previous studies found that phosphorylation of MeCP2 on either serine 80 or serine 421 is related to gene silencing or activation^38^,^39^. Stimulation of neuronal activity is associated with the loss of phosphorylation at serine 80^42^. In contrast, neuronal activity was accompanied by phosphorylation at serine 421^49^. Furthermore, knock-in mice of phospho-MeCP2-S80 or -S421 show distinct consequences *in vivo*, where MeCP2-S80 phosphorylation is associated with inhibition of certain genes and MeCP2-S421 phosphorylation is linked to gene activation^41^,^42^. More importantly, MeCP2-S80 phosphorylation is relevant to apoptosis^50^, suggesting that the phosphorylation of MeCP2-S80 and that of MeCP2-S421 may play different roles in gene regulation. This may allow an individual MeCP2 molecule to act as either a transcriptional activator or a repressor, depending on its specific modifications^41^,^51^. Taken together, our findings indicate that phospho-MeCP2-S421 might act as a regulator that participates in the interaction with VEGF in the pathogenesis of PDR upon oxidative stress and ischemia stimulation.

The development of angiogenesis requires both upregulation of angiogenetic stimulators and downregulation of angiogenesis inhibitors. We found that the significantly increased VEGF expression accompanied low PEDF expression in the PDR membranes, as demonstrated by both immunostaining and western blot. The balance between angiogenic and anti-angiogenic factors is critical in determining whether neovascularization will occur in the retina. But how the epigenetic factors affect VEGF and PEDF expression in the development of PDR are still unknown. Chao indicated that phospho-MeCP2-S80 and -S421 function like yin and yang to maintain the homeostasis of cell. Loss of balance between phospho-MeCP2-S80 and -S421 will result in cell dysfunction^38^. Our results showed that the low expression of phospho-MeCP2-S80 and the high expression of phospho-MeCP2-S421 were presented in PDR membranes, mimic hypoxia and knock down MeCP2 regulate VEGF expression, suggesting that aberrant expression of phospho-MeCP2-S80 and -S421 might play a role in retinal neovascularization, in part, by modulating the balance between angiogenic and anti-angiogenic factors such as VEGF and PEDF based on the result obtained from current study. However, further experiments, such as the effect of knock-down or overexpression of phospho-MeCP2-S421 specifically on the expression of VEGF and PEDF, the relationship of low PEDF expression to phospho- MeCP2-S80 and -S421, and the interaction of phosphorylated MeCP2-S421 specifically with VEGF and its signaling pathway are needed to answer the questions in the future.

## Conclusion

The highly expressed phospho-MeCP2-S421 and its association with VEGF in PDR membranes suggests that phospho-MeCP2-S421 might involve in the pathogenesis of PDR.

## Patients and Methods

### Pre-retinal membranes

All procedures conformed to the Declaration of Helsinki for research involving human subjects. The Institutional Review Board of Henan Eye Institute approved the use of human specimens. Informed consent was obtained from all subjects.

A total of 33 patients with PDR (33 eyes) and 11 patients (11 eyes) with IEM were included in this study for retrospective analysis. The medical records of all patients were reviewed retrospectively.

All 33 patients with PDR had undergone pars plana vitrectomy and membrane peeling in our eye hospital. Patients who had received intraocular injections of steroids or anti-VEGF drugs before surgery and those who had received laser treatment within two months prior to surgery were excluded. All patients with fibrotic vascular membranes that caused retinal detachment or with hemorrhage requiring pars plana vitrectomy were included in this study. The fibrotic vascular membranes were defined as existing erythrocytes in blood vessels within the PDR membranes or ghost vessel in the fibrotic membranes. Patients without diabetic retinopathy but who had IEMs requiring pars plana vitrectomy were included.

### Immunohistochemistry

The surgically excised membranes were fixed in 4% paraformaldehyde, embedded in paraffin wax, and cut into 3 μm sections. The sections were then stained using immunohistochemical methods after deparaffinization and rehydration (phosphate-buffered saline (PBS) pH 7.4). Antigen retrieval was performed by immersing sections of tissue in citrate buffer (pH 6.0) for 15 minutes and blocked with 5% normal goat serum for 30 min. Specimens were washed three times with after each step. Sections were incubated with anti-phosphorylated MeCP2-S80 (Thermo Fisher Scientific, Rockford, IL), anti-phosphorylated MeCP2-S421 (ABGENT, San Diego, CA) and anti-MeCP2 (non-phosphorylated form) (Thermo Fisher Scientific) and then treated by application of biotinylated secondary anti-rabbit antibody (1:400; Vector Laboratories, Burlingame, CA), and streptavidin peroxidase. The immunoreactivity was visualized using an aminoethyl carbazole kit (Zymed, South San Francisco, CA). Isotype-matched primary antibody was used as a negative control. Slides were counterstained with hematoxylin and mounted with a glycerin-gelatin medium. Retinal sections from normal adult postmortem eye were used as control. The staining intensity was scored (0 to 3) by two individuals (double-masked) as described in a previous publication^51^. Under a 40× object lens, a score of 3 indicated 70% to 100% positive cells; a score of 2 indicated 40% to 69% positive cells; a score of 1 indicated 1% to 39% positive cells; and a score of 0 indicated the absence of any staining^52^. For localization of the staining of MeCP2 and VEGF in cells of PDR membranes, large magnification of the images was obtained.

To confirm the specificity of the staining of anti-phospho-MeCP2-S421, a specific phospho-MeCP2-S421 blocking peptide (10 time over the concentration of the antibodies, ABGENT) was mixed with anti-phosphorylated MeCP2-S421 antibody (ABGENT) as the manufacturer suggested and incubated for 1 hour at room temperature. The mixture of the peptide and the antibody was applied to the section of PDR membranes. The rest of the staining steps are the same as described above.

### Double-label confocal immunofluorescence

For double labeling, the sections were first incubated with anti-phospho-MeCP2-S80, or anti-phospho-MeCP2-S421 antibodies overnight at 4 °C. The sections were incubated with a secondary antibody conjugated to fluorescein isothiocyanate for 30 min. The PBS-washed sections were then incubated with antibodies specific for VEGF (Santa Cruz Biotechnology, Santa Cruz, CA), or PEDF (R&D Systems, Inc. Minneapolis, MN); another set of double staining was performed in the same way as described above, but only phospho-MeCP2-S421 was selected for double labeling with CD31 (Abcam, Cambridge, MA), GFAP (EMD Millipore Billerica, MA) and αSMA (Sigma, St. Louis, MO) for 1 h at room temperature. The sections were washed again with PBS and then incubated with a rhodamine-conjugated secondary antibody (red color) for 30 min. The double labeling was observed by confocal laser-scanning microscopy (Nikon C1 Si, Japan). Isotype-matched primary antibody was used for negative controls. The amount of fluorescent staining was graded on a scale of 1 to 3. Intensity score of the double labeled fluorescence was established by counting the positive cell numbers in an average of three 40× objective fields in each section:−<1 cell; + = 1–5 cells; ++ = 5–20 cells; +++ = >20 cells^53^. The graders were masked to the type of membrane (PDR or IEM) during grading.

### Cell culture

Human umbilical vein endothelial cells (HUVECs) were obtained from ATCC (Manassas, VA) and were maintained culture in endothelial growth medium (EGM™ Bullet Kit, #CC-3124, Lonza, Switzerland). The cells were treated with 50 μM of Cobalt chloride (CoCl2, Sigma-Aldrich Co., MO, USA) for 3, 16 and 24 hrs, the cells then were harvested for western blot analysis. A control without CoCl2 was routinely included in each experiment.

### siRNA transfection

MeCP2 siRNA transfection was performed as previously publication^54^. Briefly, HUVECs was transfected with 10 nM MeCP2 siRNA(2 hrs) (sc-156056) or scrambled siRNA (sc-37007) (Santa Cruz Biotechnology, Dallas, TX, USA) using HiPerFect Transfection Reagent (QIAGEN) as instructed by the manufacture. 48 hrs after the transfection, the cells were harvested and protein was extracted from the cells for MeCP2 and VEGF expression by western blot analysis.

### Western blot

Paraffin tissue sections, 10 μm thick, were placed in Eppendorf tubes and deparaffinized by incubation at room temperature in xylene (Fisher Scientific, Pittsburgh, PA) and then rehydrated with a graded series of ethanol (Pharmaco Products Inc, Brookfield, IL). The rehydrated tissue sections were lysed in a RIPA buffers with the addition of a protease inhibitor cocktail (Sigma). The protein was centrifuged at 14000 × g for 20 min, and protein concentration was determined by the Bio-Rad protein assay kit (Bio-Rad, Hercules, CA). Individual proteins (MeCP2, VEGF, PEDF) were assayed in separated gel. Proteins were resolved on Tris-HCl 4–12% polyacrylamide gels (Ready Gel; Bio-Rad, Hercules, CA) at 120 V. The proteins were transferred to PVDF blotting membrane (Millipore, Bedford, MA), and the membranes were probed with antibody specific for phospho-MeCP2-S421 (ABGENT), -S80 (Active Motif), non-phospho-MeCP2 (Abcam), PEDF (R&D Systems) or VEGF (Santa Cruz). Membranes were washed and incubated with a horseradish peroxidase-conjugated secondary antibody (Vector Laboratories) for 30 min at room temperature. Images were developed by adding ECL chemiluminescence detection solution (Amersham Pharmacia Biotech, Cleveland, OH). After stripping, the membranes were re-probed with anti-GAPDH antibody (Millipore) for protein loading control. Semi-quantitation of protein expression was determined by densitometry analysis using Image J software (NIH).

### Statistical analysis

Immunostaining scores were analyzed by Fisher’s exact test; the densitometry of western blot and double-labeled confocal immunofluorescence images were analyzed by Student’s-T test. A *P* value <0.05 was accepted as significant.

## Additional Information

**How to cite this article**: Li, X. *et al*. The significance of the increased expression of phosphorylated MeCP2 in the membranes from patients with proliferative diabetic retinopathy. *Sci. Rep.* **6**, 32850; doi: 10.1038/srep32850 (2016).

## Supplementary Material

Supplementary Information
