# Supplementary material for: The significance of the increased expression of phosphorylated MeCP2 in the membranes from patients with proliferative diabetic retinopathy
Source: Sci Rep. 2016 Sep 12;6:32850. doi: 10.1038/srep32850 (PMC5018725; doi:10.1038/srep32850)
Supplement: Supplementary Information [file srep32850-s1.doc]

**The significance of the increased expression of phosphorylated MeCP2 in the membranes from patients with proliferative diabetic retinopathy**

Xiaohua Li*^1,2,3,4^, Xiaohui Liu ^1,2,3,4^, Haoyi Guo, ^1,2,3,4^ Zhaoxia Zhao ^1,2,3,4^, Yun Sui Li ^1,2,3,4^, Guoming Chen ^1,2,3,4^

^1^Henan eye Institute, Henan key laboratory of keratopathy; ^2^Henan Eye Hospital; ^3^Department of Ophthalmology, Henan Provincial People's hospital; ^4^Department of Ophthalmology, Zhengzhou University People’s hospital

Financial support: this work is supported by the National Nature Science Foundation of China (Grant #81100650) and International Scientific and Technological Cooperation Project of Technological Research and Development Project in Zhengzhou City (131PGJHZ434)

*Corresponding author: Xiaohua Li, MD, Ph.D.

NO.7 Wei Wu Road,

Zhengzhou, 450003, China

Phone: 0371-65580390

Fax: 0371-65952907

Email: [xhl_6116@163.com](mailto:xhl_6116@163.com)

**Supplementary Material**

**Supplementary information contains: Supplementary Table, Supplementary Figures and Legends**.

**Supplementary table 1.**

**
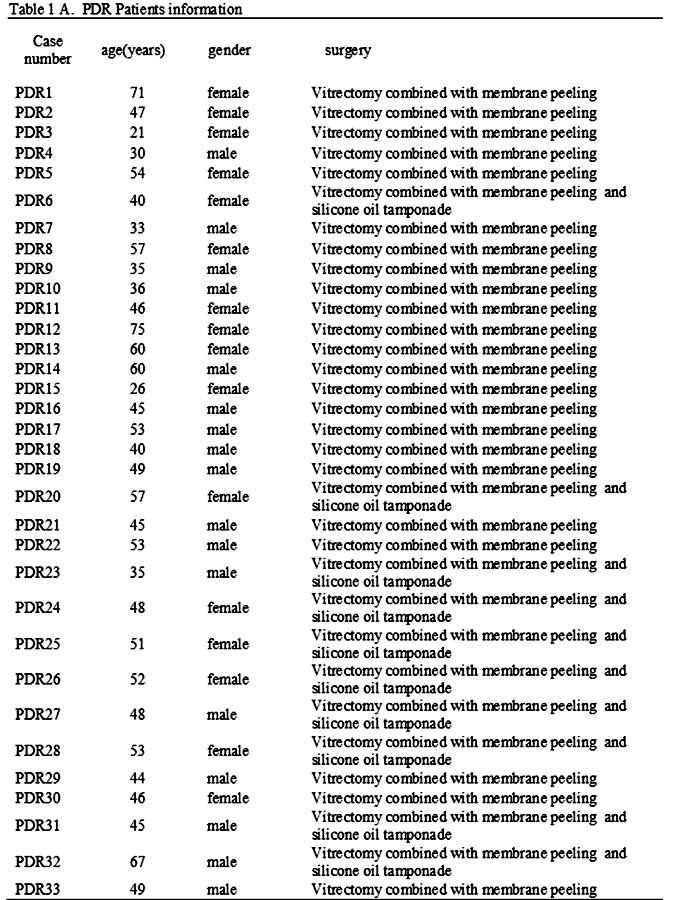
**


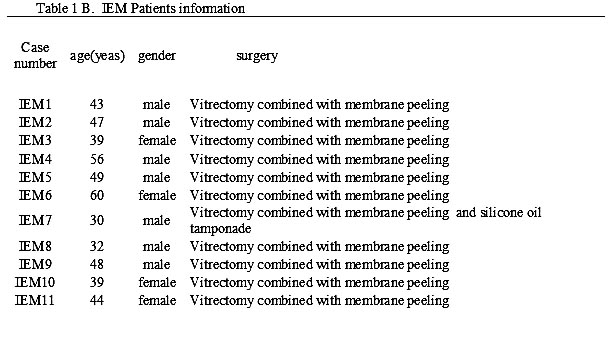


There were17 men and 16 women included in the PDR patients (A), the average age of the patients are 47.6 years, their age range was from 21-75 years. Of 11 patients with idiopathic epiretinal membrane (IEM), there are 7 men and 4 women included in the study, the average ages of the patients are 44 years, and their age range was from 30-60 years. No difference in gender distribution was demonstrated either in the patients with PDR or IEM.

**Supplementary table2.** Scores of the staining of phospho-MeCP2 S80 and S421 in the membranes of PDR and IEM.

Stained marker PDR (33) IEM (11) P*

**Score Score**

**- 1+ 2+ 3+ - 1+ 2+ 3+**

phospho-

MeCP2 80 0 30(91%) 3(9%) 0 0 10(91%) 1(9%) 0 0.20

phospho-

MeCP2 421 0 1 (3%) 4(12%) 28(85%) 2(18%) 9 (82%) 0 0 <0.001

P* <0.001 1.00

The expression of phospho-MeCP2-S80 and phospho-MeCP2-S421 in PDR and IEM membranes determined by immunohistochemistry. The immunostaining intensity was scored from 0, 1, 2, and 3. There was significant difference in the expression of phospho-MeCP2-S421 between PDR and IEM (*P*<0.001); the expression of phospho-MeCP2-S421 also was significantly higher than that of phospho-MeCP2-S80 in PDR (*P*<0.001).*Fisher’s Exact Test p-values, where *P*<0.05 is statistically significant.

**Supplementary table 3**. Scores of the double labeling of phospho-MeCP2 S80 or S421 with VEGF and PEDF in PDR membranes

Average score + SD P *

phospho-MeCP2 S80+VEGF 1.3+0.2

phospho-MeCP2 S80+PEDF 1.2+0.15 >0.05

phospho-MeCP2 S421+ VEGF 2.75+0.3

phospho-MeCP2 S421+ PEDF 0.8 + 0.2 <0.01

The intensity of double labeling of phospho-MeCP2-S80 and phospho-MeCPS2-421 with VEGF or PEDF in PDR membranes determined by immunofluorescence staining. The intensity was scored from 0, 1, 2, 3. VEGF is significantly colocalized with phospho-MeCP2-S421 (*P*<0.01); There is no significant difference in the double labeling of MeCP2 with either VEGF or PEDR (*P*>0.05). *Student *T-test*

**Supplementary Fig 1**

**
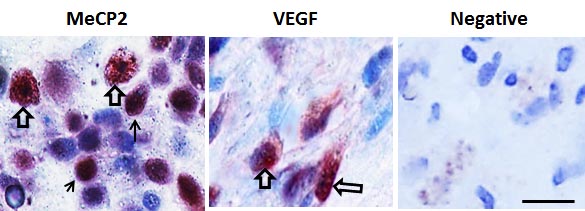
**

The expression of MeCP2 and VEGF in the cells of PDR membranes with large magnification as demonstrated by immunohistochemistry. Open arrows indicate the cytoplasm staining of MeCP2 and VEGF, where the solid arrows show the immnoreactivity of MeCP2 in nuclei. Scale bar: 100um. Original magnification, 1000x

**Supplementary Fig2**

**
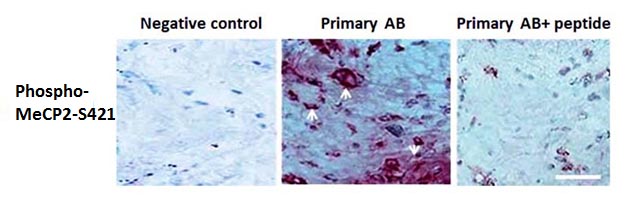
**

The effects of the blocking peptide on the specificity of the staining of anti- phospho-MeCP2-S421. Left panel: negative control without primary antibody; Middle panel: with the addition of anti- phospho-MeCP2-S421 antibody, showing abundant immunoreactivity; right panel: incubation of the mixture of the blocking peptide with phospho-MeCP2-S421antibody, the positive staining of the phospho-MeCP2-S421 is abolished comparing with the middle panel. Scale bar: 50µm. Original magnification, 400x.

**Supplementary Fig3**


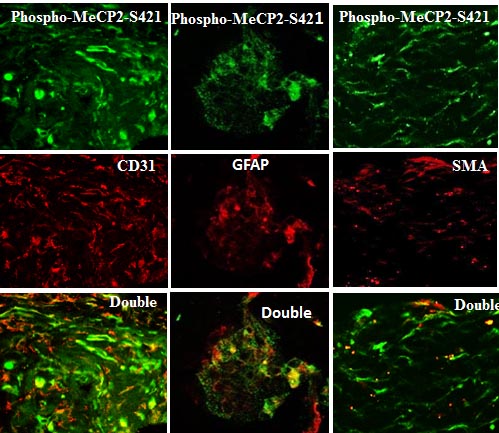


Phospho-MeCP2-S421 double labeling with CD31, GFAP and αSMA in human PDR membrane. The phospho-MeCP2-S421 was stained as green. CD31, GFAP and αSMA were stained in in red. Yellow shows co-localization of phospho-MeCP2-S421 with CD31 or GFAP or αSMA. Scale bar: 100 µm. Original magnification, 200x.

**Supplementary Fig 4**

**
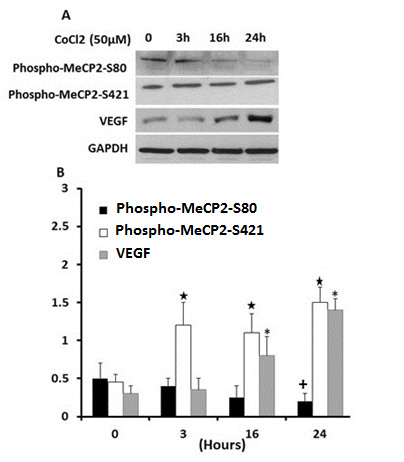
**

The effects of CoCl2 on the expression of phospho-MeCP2-S80,S421and VEGF in HUCAC cells. (A) The extracted proteins were used in immunoblotting to detect phospho-MeCP2-S80, S421and VEGF .GAPDH was used as protein loading control. Blot is representative. (B)Relative levels of phospho- MeCP2-S80, S421and VEGF from 3 independent experiments were quantified by measuring band intensity with Image J software. Compared with control **+**
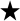
*P< 0.05.

**Supplementary Fig 5.**


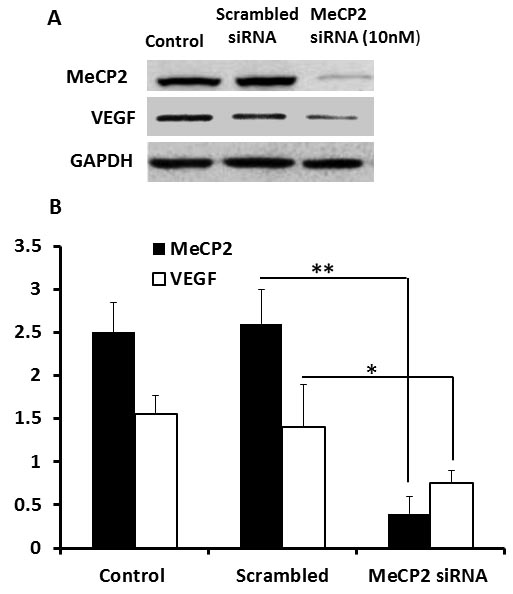


The effects of knock down MeCP2 using siRNA on the expression of MeCP2 and VEGF in HUCAC cells. (A) The extracted proteins were used in immunoblotting to detect MeCP2 and VEGF. GAPDH was used as protein loading control. Blot is representative. (B) Relative levels of MeCP2 and VEGF from 3 independent experiments were quantified by measuring band intensity with Image J software. Compared with scrambled *P< 0.05. **P<0.01.
